# Supplementary material for: Natural leaf shape variation reveals diverse transcriptional targets of GmJAG1 during soybean leaf development
Source: Plant J. 2026 Aug 26;127(4):e71088. doi: 10.1111/tpj.71088 (PMC13517971; doi:10.1111/tpj.71088)
Supplement: Supplementary file 1 [file TPJ-127-0-s002.docx]

**Supplemental Figures.**

**Figure S1. Extended RNA-seq quality control and batch correction assessment. (A)** Library size (total sequencing reads) for all 60 RNA-seq samples, grouped by developmental timepoint (TP1-TP5). Within each timepoint, bars represent individual biological replicates for each of the four soybean genotypes (color-coded). The dashed horizontal line indicates the overall mean sequencing depth (29.5 million reads). **(B)** Relative Log Expression (RLE) plots before (left) and after (right) ComBat-seq batch correction. Each boxplot represents the distribution of expression deviations from the gene-wise median for one sample. Samples are colored by sequencing batch. The dashed horizontal line indicates the expected median of zero. **(C)** Comparison of mean sample correlations within and between sequencing batches before and after ComBat-seq correction. **(D)** Hierarchical clustering dendrogram of all RNA-seq samples after ComBat-seq batch correction. Samples were clustered using average linkage based on Pearson correlation distances (1- r). Colored bars below the dendrogram indicate developmental timepoint (TP1-TP5), leaf type (Broad, Narrow), and sequencing batch (1, 2). Samples cluster primarily by developmental stage rather than batch, indicating effective batch correction.

**Figure S2. Temporal differential gene expression profiles by individual genotype.** **(A)** Temporal expression persistence of *JAG1* targets. Pie chart showing that 99.1% of candidate targets (n = 1,553) maintained differential expression throughout development (blue), while only 0.9% (n = 14) showed expression differences restricted to early timepoints (red). **(B)** Diverging bar plots showing the number of differentially expressed genes at each developmental timepoint (TP2-TP5) relative to the shoot apex (TP1) for each of the four soybean genotypes used in this study. Two narrow-leaflet genotypes (PI 547745 and PI 612713B) and two broad-leaflet genotypes (LD11-2170 and PI 532462A) are shown. Red bars indicate upregulated genes and blue bars indicate downregulated genes (|log₂FC| > 1, FDR < 0.05).

**Figure S3. *GmJAG1* and *GmJAG2* protein sequence alignment.** Pairwise alignment of *GmJAG1* (*Glyma.20G116200*, 256 aa) and *GmJAG2* (*Glyma.10G273800*, 257 aa) showing 91.1% amino acid identity. Protein sequences were translated from coding sequences deposited in GenBank: *JAG1* (JX119212.1) and *JAG2* (JX119214.1) from Jeong et al. (2012). Functional domains are annotated: EAR motif (*, position 8-14), nuclear localization signal (@, position 36-39), C2H2 zinc finger (#, position 52-82), and proline-rich region (~, position 141-151). Both paralogs retain identical EAR motif (LDLNNLP) and C2H2 zinc finger domains including the QALGGH DNA-binding motif. Alignment performed using Needleman-Wunsch algorithm with BLOSUM62 matrix.

**Figure S4. Single-cell expression of *JAG1* pathway components across SAM cell-type clusters.** **(A)** *GmJAG1* percent expressing across 16 SAM cell-type clusters from Fan et al. (2025) snRNA-seq data (9,869 nuclei). LP1 = Leaf Primordium 1 (515 cells, red). **(B)** Mean percent expressing for *TPR* co-repressors (12 genes) and HDAC complex (11 genes) across all 16 SAM clusters*.* **(C)** Seurat-style dot plot of nine individual *KRP* genes across 16 clusters. Dot size represents percent of cells expressing; dot color represents per-gene scaled mean expression. **(D)** Seurat-style dot plot of seven *JAG1*-target cyclin genes across 16 clusters. Dot size and color encoding as in (C).

**Figure S5. Phylogenetic analysis of D-type cyclins across four plant species.** Maximum likelihood phylogeny of 70 D-type cyclin protein sequences from *Glycine max* (39 sequences), *Arabidopsis thaliana* (10), *Medicago truncatula* (15) and *Oryza sativa* (6 representatives, one per subclade). The tree is rooted on *O. sativa* *CYCD3*. Six conserved subclades (*CYCD1, CYCD2/4, CYCD3, CYCD5, CYCD6, CYCD7*) are indicated by colored background shading and labeled vertical bars. Red asterisks mark the two *GmJAG1*-regulated D-type cyclins in the *CYCD1* and *CYCD6* subclade. Blue diamond marks *AtCYCD3;3*, the Arabidopsis *JAG* target.

**Figure S6. qRT-PCR validation of RNA-seq findings.** Correlation between RNA-seq log₂ fold change and qRT-PCR log₂ fold change for eight genes validated across four soybean genotypes at TP1 (shoot apex). Each point represents the mean of two pairwise comparisons (PI 612713B vs PI 532462A and PI 547745 vs LD11-2170). Gene names are italicized. The dashed grey line indicates 1:1 identity; the blue line shows linear regression with 95% confidence interval. NPH3 (diamond) was undetectable by qPCR in PI 532462A; fold change was estimated using a Cq floor of 40. Pearson correlation coefficient, P-value, and regression slope are shown. Expression was normalized to the geometric mean of Actin 11 and RPL30 reference genes.

**Figure S7. Selection of the soft-thresholding power for WGCNA. (A)** Scale-free topology model fit (signed R²) versus soft-thresholding power (β). The dashed line marks the R² = 0.80 criterion; β = 5 (orange) was the lowest power achieving a fit above this threshold (R² = 0.81). **(B)** Mean connectivity versus soft-thresholding power; mean connectivity at β = 5 was 832. The network was built from 28,905 genes across 60 samples using a signed-hybrid topology and biweight midcorrelation.
